# Vaccinia-induced epidermal growth factor receptor-MEK signalling and the anti-apoptotic protein F1L synergize to suppress cell death during infection

**DOI:** 10.1111/j.1462-5822.2009.01327.x

**Published:** 2009-05-26

**Authors:** Antonio Postigo, Morag C Martin, Mark P Dodding, Michael Way

**Affiliations:** Cancer Research UK London Research Institute44 Lincoln's Inn Fields, London WC2A 3PX, UK.

## Abstract

F1L is a functional Bcl-2 homologue that inhibits apoptosis at the mitochondria during vaccinia infection. However, the extent and timing of cell death during ΔF1L virus infection suggest that additional viral effectors cooperate with F1L to limit apoptosis. Here we report that vaccinia growth factor (VGF), a secreted virulence factor, promotes cell survival independently of its role in virus multiplication. Analysis of single and double knockout viruses reveals that VGF acts synergistically with F1L to protect against cell death during infection. Cell survival in the absence of F1L is dependent on VGF activation of the epidermal growth factor receptor. Furthermore, signalling through MEK kinases is necessary and sufficient for VGF-dependent survival. We conclude that VGF stimulates an epidermal growth factor receptor-MEK-dependent pro-survival pathway that synergizes with F1L to counteract an infection-induced apoptotic pathway that predominantly involves the BH3-only protein Bad.

## Introduction

Upon virus infection most cells attempt to undergo cell death as a means to limit viral spread. To circumvent this, viruses have evolved numerous mechanisms to inhibit apoptosis, thereby prolonging the survival of infected cells to facilitate productive replication (Roulston *et al*., 1999). Consequently, many viruses encode sequence homologues of anti-apoptotic members of the Bcl-2 family (Cuconati and White, 2002). Like their cellular counterparts, these viral proteins inhibit the pro-apoptotic Bak and Bax from executing mitochondrial-dependent cell death. Bcl-2 sequence homologues are noticeably absent from most poxvirus genomes. However, the Lepripox virus myxoma encodes a protein, M11L, which prevents apoptosis by inhibiting Bak and Bax (Wang *et al*., 2004; Su *et al*., 2006). Similarly, vaccinia virus encodes a mitochondrially targeted protein, F1L, which is able to inhibit apoptosis by binding and preventing activation of Bak and Bim (Wasilenko *et al*., 2005; Postigo *et al*., 2006; Taylor *et al*., 2006). This anti-apoptotic activity is dependent on a BH3-like, Bak-binding domain, suggesting that F1L acts as a functional Bcl-2 homologue (Postigo *et al*., 2006).

In the absence of F1L cells succumb to cell death much earlier than during infection with Western Reserve (WR), even though replication and spread appear unaffected (Wasilenko *et al*., 2005; Postigo *et al*., 2006). Furthermore, it has been reported that infection triggers a cellular suicide response shortly after virus entry (Ramsey-Ewing and Moss, 1998) and before F1L is detected at 2–3 h after infection (Postigo *et al*., 2006). Given this, we wondered whether an additional early viral protein cooperates with F1L to prevent cell death. One possible candidate expressed early during infection is the highly conserved vaccinia growth factor (VGF), an epidermal growth factor (EGF)-like growth factor (Brown *et al*., 1985). EGF belongs to a family of ligands that is conserved from invertebrates to mammals (Yarden and Sliwkowski, 2001). All these ligands induce ligand-induced homo- and heterodimerization of the ErbB1-4 receptor tyrosine kinases leading to activation of Ras-MEK-ERK, phospholipase C and STAT pathways (Yarden and Sliwkowski, 2001). EGF receptor (EGFR) (ErbB1)-mediated signalling stimulates cell proliferation and differentiation, but it can also act to inhibit apoptosis (Sibilia *et al*., 2000; Jost *et al*., 2001a; Yarden and Sliwkowski, 2001).

Vaccinia growth factor is secreted early during infection. It binds EGFR homodimers with reduced affinity, a feature that enhances signalling by attenuating receptor inactivation (Tzahar *et al*., 1998). Deletion of VGF from the vaccinia genome (ΔVGF) leads to reduced plaque size *in vitro* and reduced virulence *in vivo*, consistent with a role in virus replication (Buller *et al*., 1988a; b). Furthermore, secreted VGF acts as a mitogen that primes non-infected cells for subsequent infection (Buller *et al*., 1988a; b). In addition to this non-cell autonomous effect, VGF activates ERK in infected cells, which is required for efficient viral replication and multiplication (Andrade *et al*., 2004).

In this study we report that VGF promotes survival of vaccinia-infected cells by inducing an EGFR-MEK-dependent pathway that mimics host survival signals in epithelial cells. Furthermore, by using the ΔF1L-sensitized genetic background we show that VGF and F1L act synergistically to counteract a cell death pathway predominantly dependent on the BH3-only protein Bad.

## Results

### VGF and F1L act synergistically to prevent infection-induced cell death

**Fig. 1:**
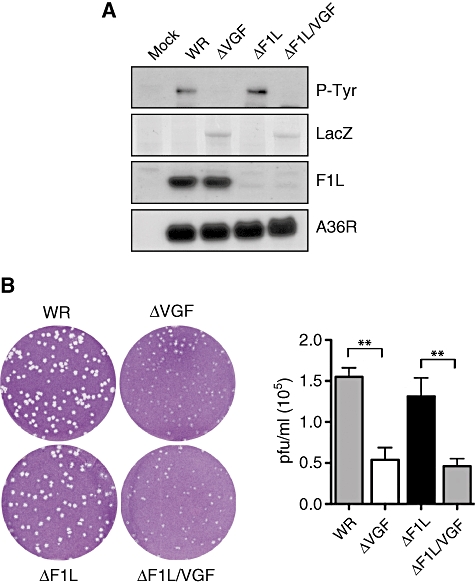
Loss of F1L does not reduce ΔVGF plaque size or virus production. A. Western blot analysis of HeLa cell lysates infected with the indicated virus strains for 20 h post infection reveals that viruses encoding VGF phosphorylate the EGFR (P-Tyr). EGFR is not activated by ΔVGF and ΔF1L/VGF, which contain a LacZ reporter in the VGF loci (Ponceau staining). B. Representative images of plaque formation by the indicated virus strains in BSC-1 cells visualized with crystal violet 4 days post infection. The graph shows quantitative analysis of infectious virus (plaque forming units ml^−1^) present in the culture supernatant of the indicated virus strains 24 h post infection determined using standard plaque assays on BSC-1 cells. The error bars represent standard error of the mean and ** indicates *P* < 0.01.

In order to test whether growth factor signalling by VGF contributes to cell survival during infection, we deleted the F1L gene from the ΔVGF virus. Infection with the resulting double mutant virus (ΔF1L/VGF) leads to inhibition of phosphorylation of the EGFR, indicating that the virus, like ΔVGF, was unable to induce growth factor signalling (Fig. 1A). Consistent with previous observations using the single-deletion strains, we found that WR and ΔF1L infection produces large plaques, while both the ΔVGF and ΔF1L/VGF viruses form small plaques (Fig. 1B). Re-infection assays with WR, ΔVGF, ΔF1L or ΔF1L/VGF viruses confirmed that the absence of VGF results in a reduction in infectious particle production consistent with the plaque phenotype and the replication defect caused by the absence of VGF (Fig. 1B). Thus, absence of F1L in the double mutant does not exacerbate the replication defect reported for the ΔVGF virus.

**Fig. 2:**
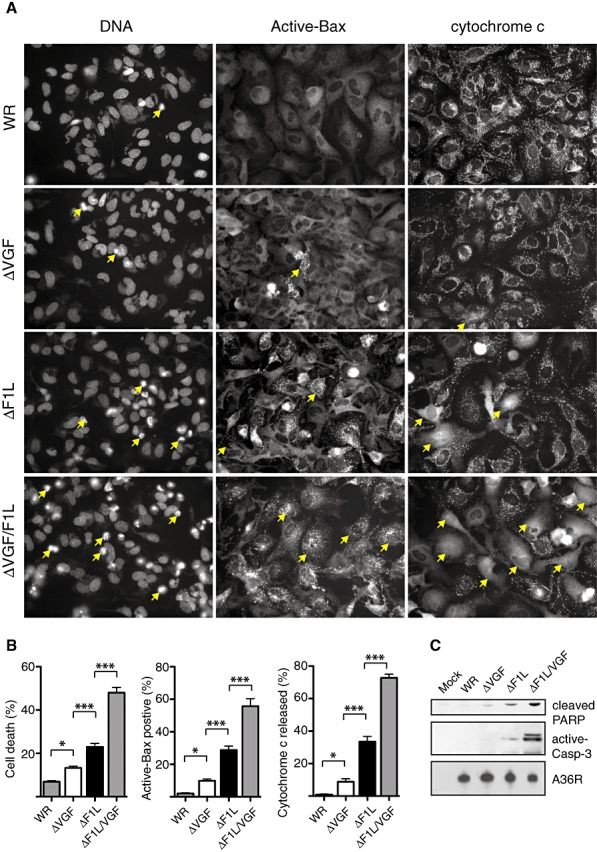
Loss of VGF increases cell death in the absence of F1L. A. Representative immunofluorescence images illustrating the changes in apoptosis status during infection with the indicated virus at 20 h post infection. Yellow arrowheads indicated DNA fragmentation (DNA, left column), Mitochondrial Bax activation (Active-Bax, middle column) and release of cytochrome c from mitochondria (cytochrome c, right column). B. Quantification of cell death in HeLa cells infected with the indicated virus at 20 h post infection. Error bars represent standard error of the mean, *P< 0.05 and ***P< 0.001. C. Western blot analysis of infected cells at 20 h post infection indicates that there is increasing amounts of processed PARP and cleaved caspase-3 during ΔVGF, ΔF1L and ΔF1L/VGF infections.

To determine if VGF also promotes cell survival, we examined whether infection with the ΔVGF virus resulted in increased apoptosis using quantitative immunofluorescence assays that detect increases in fragmented nuclei, accumulation of activated Bax and loss of mitochondrial cytochrome C (Fig. 2A). All three assays revealed a small but significant increase in apoptosis after ΔVGF infection (Fig. 2B). A more pronounced level of cell death was observed in ΔF1L infection cells (Fig. 2B). Moreover, infection with the ΔF1L/VGF virus resulted in a synergistic increase in cell death in all three assays (Fig. 2B). The increase in cell death in the absence of VGF or F1L also correlated with classical biochemical hallmarks of apoptosis (Fig. 2C). Infection with the WR did not induce poly (ADP-ribose) polymerase (PARP) cleavage or activation of caspase-3. Loss of VGF resulted in a small but detectable cleavage of PARP and caspase-3 activation, while deletion of F1L induced pronounced PARP cleavage and activation of caspase-3. Cleavage of these apoptotic markers was significantly enhanced in the absence of both F1L and VGF during ΔF1L/VGF infections (Fig. 2C). Taken together, our results show that infection with the ΔF1L/VGF virus leads to a synergistic increase in cell death, consistent with a role for VGF in promoting cell survival during infection.

### VGF mediates cell survival in an EGFR-dependent manner

**Fig. 3:**
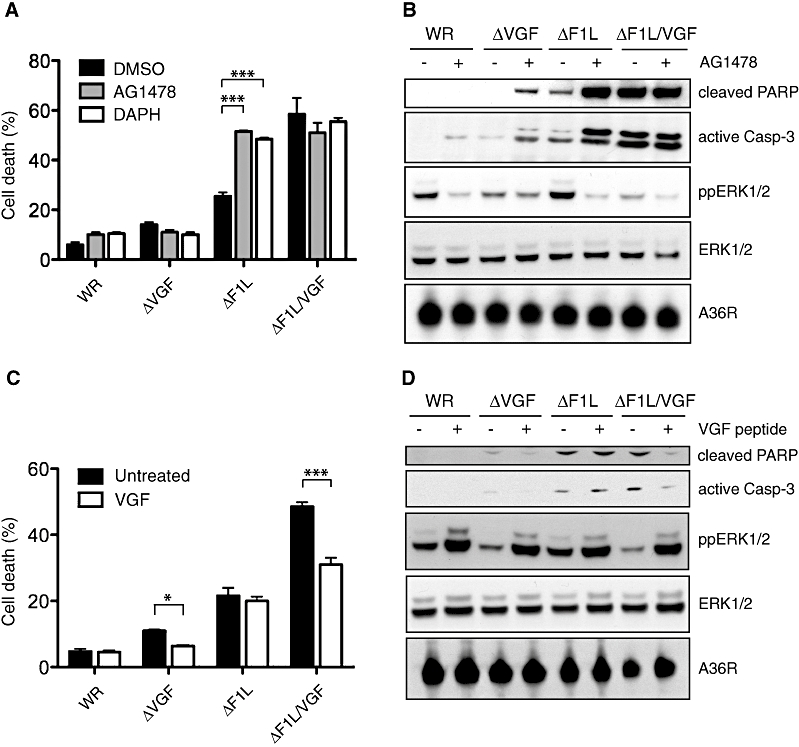
VGF mediates cell survival via the EGFR. A. Quantitative analysis of DNA fragmentation in HeLa cells infected for 20 h with the indicated virus strains in the presence or absence of the EGFR inhibitors AG1478 and DAPH. B. Western blot analysis of cells infected for 20 h with the indicated virus in the presence (+) or absence (−) of AG1478 reveals that loss of EGFR signalling reduces ERK phosphorylation and increases PARP processing as well as caspase-3 cleavage. C. Quantitative analysis of cell death in HeLa cells infected with indicated virus strains for 20 h in the presence or absence of the VGF peptide. Error bars represent standard error of the mean, *P< 0.05 and ***P< 0.001. D. Western blot analysis of infected cells in the presence (+) or absence (−) of the VGF peptide reveals that activation of EGFR signalling promotes ERK phoshorylation and reduces PARP processing and caspase-3 cleavage during ΔVGF and ΔF1L/VGF infection.

It has been reported that VGF preferentially binds EGFR-containing hetero- and homodimers. To establish whether VGF stimulates cell survival solely via the EGFR receptor, we examined the effect of AG1478, a specific EGFR inhibitor, and DAPH, an inhibitor of ErbB1/2, on the survival of HeLa cells infected with WR, ΔVGF, ΔF1L and ΔF1L/VGF viruses (Fig. 3A and B). Western blot analysis revealed that AG1478 inhibited activation of the ERK pathway due to WR infection (Fig. 3B). Both AG1478 and DAPH treatment substantially increased cell death during ΔF1L infection to levels that were similar to those observed with the ΔF1L/VGF virus (Fig. 3A). This correlates with AG1478 blockade of vaccinia-induced EGFR signalling and downstream ERK activation. In addition, activation of ERK by phosphorylation is severely reduced in the absence of VGF (Fig. 3B). Furthermore, similar to the corresponding cell death counts, analysis of apoptosis markers revealed an increase in PARP and caspase-3 cleavage in lysates from ΔF1L-infected cells to the levels observed with ΔF1L/VGF (Fig. 3B). AG1478 had no additional effects on PARP and caspase-3 cleavage in cells infected with the ΔF1L/VGF virus (Fig. 3B).

Having established that VGF-mediated survival depends on the EGFR, we next asked whether exogenously supplied VGF is sufficient to rescue F1L-independent cell survival during ΔF1L/VGF infection. Addition of VGF led to an increase in cell survival in ΔVGF-infected cells, but had no effect on ΔF1L-infected cells (Fig. 3C). Moreover, VGF decreased cell death in ΔF1L/VGF-infected cells to levels similar to ΔF1L (Fig. 3C). Addition of the synthetic VGF peptide also stimulated ERK phosphorylation for the duration of the experiment (Fig. 3D). Furthermore, VGF application inhibited PARP and caspase-3 cleavage in ΔF1L/VGF-infected cells (Fig. 3D). Together, our results demonstrate that VGF signalling via the EGFR is sufficient to promote F1L-independent cell survival during infection.

### Late gene expression is dispensable for VGF-mediated cell survival

**Fig. 4:**
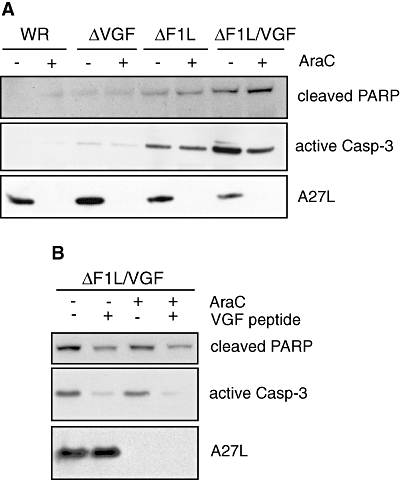
VGF stimulates cell survival independent of late gene expression. A. Western blot analysis of infected cells in the presence (+) or absence (−) of AraC reveals that PARP processing and caspase-3 cleavage is unaffected by inhibition of late gene expression. AraC-mediated inhibition of late gene expression was confirmed by the absence of A27L, a late viral protein. B. Western blot analysis of ΔF1L/VGF-infected cells in the presence (+) or absence (−) of the VGF peptide reveals that the reduction in PARP processing and caspase-3 cleavage by VGF is not inhibited by the presence (+) of AraC.

Expression of VGF is required for efficient replication and late gene expression. It is possible therefore that VGF mediates cell survival indirectly, for instance, if it is required for expression of a late gene that promotes cell survival. To exclude whether this is the case, we infected cells with the four different viruses in the presence of cytosine arabinoside (AraC), an inhibitor of DNA replication and late gene expression. AraC would be expected to exacerbate cell death if VGF survival depends on late gene expression. However, Western blot analysis of infected cell lysates treated with AraC showed no further increase in PARP cleavage or caspase-3 processing in any of the virus strains (Fig. 4A). This suggests that VGF, as well as F1L exert their effects on cell survival independently of late gene expression.

Addition of exogenous VGF peptide is sufficient to limit cell death during ΔF1L/VGF infection to the levels observed with ΔF1L (Fig. 3C). Addition of AraC did not increase PARP and caspase-3 cleavage in ΔF1L/VGF-infected cells in the presence of the VGF peptide (Fig. 4B). These experiments using an exogenously added peptide demonstrate that VGF promotes survival independently of late gene expression.

### MEK is necessary and sufficient to mediate VGF-dependent survival

**Fig. 5:**
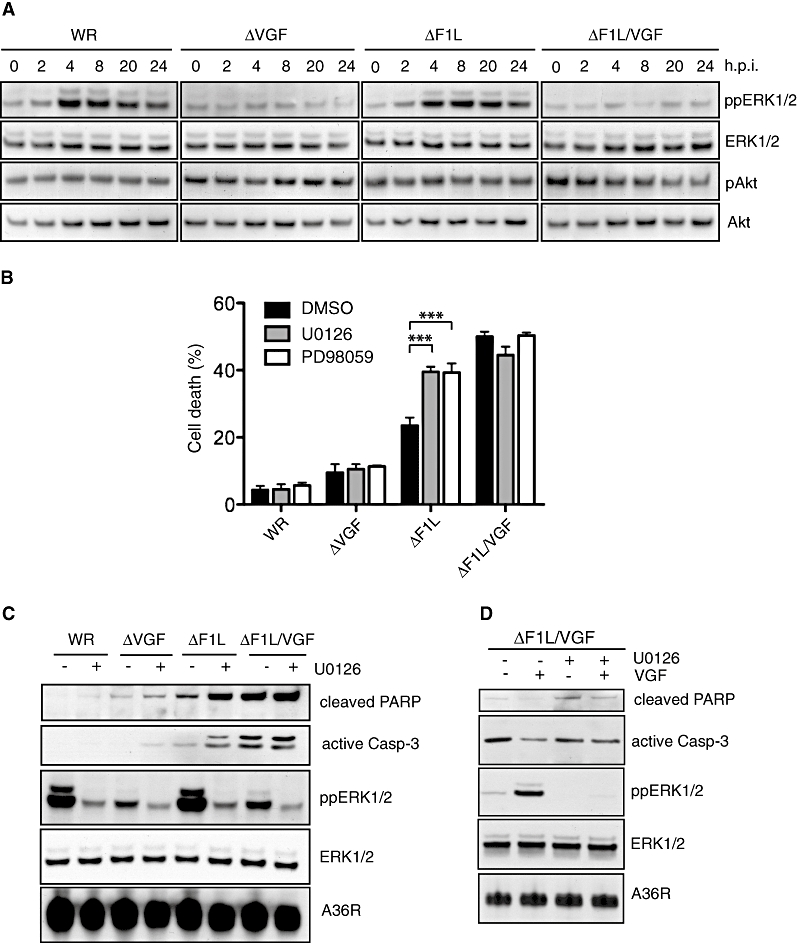
Activated ERK is necessary for VGF-dependent cell survival. A. Western blot analysis of HeLa cells infected with indicated virus reveals that ERK is phosphorylated during WR and ΔF1L but not ΔVGF and ΔF1L/VGF infection. The status of Akt phosphorylation remains relatively constant throughout infection with all strains. B. Quantitative analysis of cell death in HeLa cells infected with indicated virus in the presence or absence of the MEK1/2 inhibitor UO126. Error bars represent standard error of the mean and ***P< 0.001. C. Western blot analysis of infected cells in the presence (+) or absence (−) of UO126 reveals that loss of MEK1/2 activity results reduced ERK phosphorylation and an increase in processed PARP and cleaved caspase-3 in ΔF1L infected cells. D. Western blot analysis of ΔF1L/VGF-infected cells in the presence (+) or absence (−) of VGF peptide reveals that peptide-mediated reduction in PARP processing and caspase-3 cleavage is inhibited by the presence (+) of UO126.

Activation of the EGFR induces cell survival by stimulating the Ras-MEK-ERK and phosphoinositol 3-phosphate kinase (PI3K)-Akt pathways. ERK is activated by phosphorylation in response to vaccinia virus infection, and this increase in phosphorylation is severely reduced in the absence of VGF (Figs 3B and 5A). In contrast, we failed to see changes in Akt phosphorylation throughout infection irrespective of the viral strain (Fig. 5A). We therefore investigated whether the Ras-MAPK pathway contributes to VGF-dependent survival.

To determine if VGF-mediated survival is dependent on activation of the Ras-MAPK pathway, we examined the effect of the specific MEK1/2 inhibitors UO126 and PD98059 during infection with WR and the deletion strains. Treatment with either drug had no effect on ΔVGF or ΔF1L/VGF-induced cell death, but increased ΔF1L-induced cell death to similar levels as observed with the ΔF1L/VGF virus (Fig. 5B). WR and ΔF1L-mediated ERK activation was blocked in the presence of UO126 (Fig. 5C). Western blot analysis of the extent of PARP and caspase-3 cleavage confirmed that inhibition of MEK1/2 by UO126 resulted in increased apoptosis in ΔF1L infected cells (Fig. 5C). Conversely, addition of VGF peptide to ΔF1L/VGF-infected cells activated ERK and at the same time reduced PARP and caspase-3 cleavage (Fig. 5D). The addition of U0126 blocked ERK phosphorylation by VGF peptide and abrogated the VGF-mediated decrease in ΔF1L/VGF-induced PARP and caspase-3 cleavage (Fig. 5D).

**Fig. 6:**
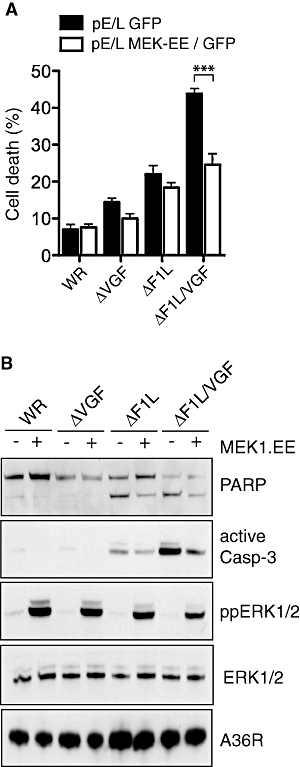
Activated ERK is sufficient for VGF-dependent cell survival. A. Quantitative analysis of cell death in HeLa cells 20 h post infection with indicated virus in the presence or absence of constitutively active MEK1/2 (MEK-EE). Error bars represent standard error of the mean and ***P< 0.001. B. Western blot analysis of infected cells in the presence (+) or absence (−) of MEK-EE reveals that activation of MEK1/2 increases ERK phosphorylation in all strains and decreases PARP processing as well as caspase-3 cleavage during ΔF1L/VGF infection.

We next examined if constitutively active MEK (MEK-EE) would be sufficient to promote F1L-independent cell survival during infection in the absence of an upstream VGF signal. To ensure that cell death was only quantified in infected HeLa cells expressing MEK-EE we used GFP expression as a co-reporter for cell transfection. This analysis reveals that expression of MEK-EE did not impact on ΔF1L-induced death (Fig. 6A). In contrast, cell death in ΔF1L/VGF-infected cells was reduced to similar levels as observed in ΔF1L infections (Fig. 6A). Western blot analysis revealed that there was a corresponding decrease in infection-induced PARP and caspase-3 cleavage in ΔF1L/VGF cells expressing MEK-EE (Fig. 6B). Our data show that activation of MEK downstream of the EGFR is necessary and sufficient for the VGF–mediated cell survival during infection.

### VGF and F1L cooperatively block infection-induced cell death via Bad

**Fig. 7:**
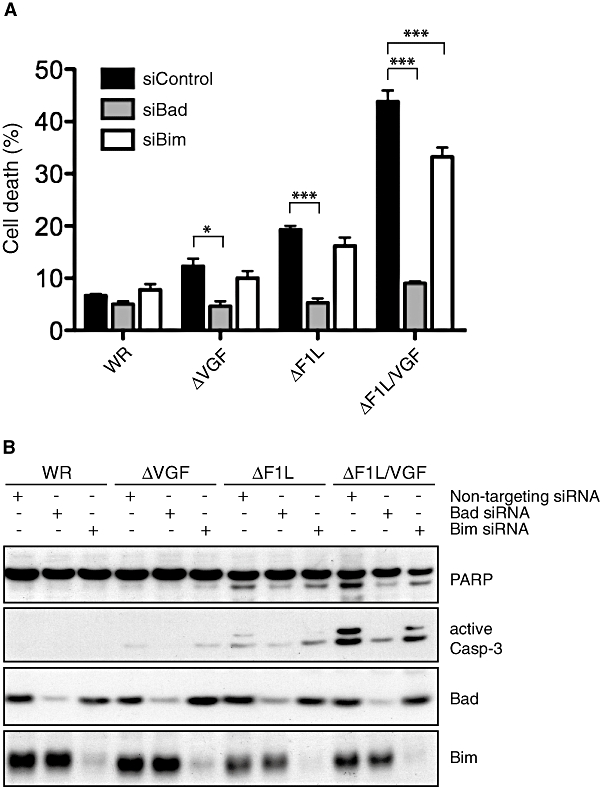
Infection-induced cell death is principally dependent on Bad. A. Quantitative analysis of cell death in HeLa cells transfected with the indicated siRNA against Bim and Bad at 20 h post infection. Error bars represent standard error of the mean, *P< 0.05 and ***P< 0.001. B. Western blot analysis of cells infected with the indicated virus after RNAi-mediated knockdown of Bim or Bad reveals that loss of Bad results in reduced PARP processing and caspase-3 cleavage.

It is at present unclear what molecular trigger induces vaccinia-induced cell death when either F1L or VGF are absent. If VGF and F1L synergize to prevent cell death, the loss of a crucial host component mediating cell death should abrogate cell death in ΔF1L/VGF cells. Bad and Bim are two BH3-only pro-apoptotic members of the Bcl-2 family that are known to be regulated by growth factor signalling (Puthalakath and Strasser, 2002). We therefore tested whether Bad and Bim might be responsible for mediating infection-induced cell death by examining the effect of their downregulation through RNAi-mediated ablation (Fig. 7). Remarkably, for the three deletion viruses, we found that loss of Bad resulted in a dramatic reduction in cell death to similar levels to that observed in WR infections (Fig. 7A). In contrast, knockdown of Bim only resulted in a significant reduction in cell death in ΔF1L/VGF-infected cells. These results were consistent with Western blot analysis of apoptosis markers (Fig. 7B). Loss of Bad significantly reduced PARP and caspase-3 cleavage in both ΔF1L and ΔVGF-infected cells. In ΔF1L/VGF-infected cells loss of Bim resulted in a small reduction of cell death, whereas lack of Bad resulted in a large reduction in PARP and caspase-3 cleavage (Fig. 7). Our data demonstrate that VGF and F1L cooperate to suppress vaccinia-induced cell death through a pathway that primarily involves Bad.

## Discussion

The EGF stimulates complex signalling networks to promote proliferation, differentiation, migration or survival in a context-dependent manner (Yarden, 2001; Yarden and Sliwkowski, 2001). Consistent with its critical role in cell proliferation and survival components of the EGF signalling networks are frequently constitutively activated within tumours (Olayioye *et al*., 2000). It is not surprising given that viruses are also dependent on cell survival for productive replication that they have evolved elegant mechanisms to inhibit infection-induced cell death (Roulston *et al*., 1999). Our data now clearly show that vaccinia virus stimulates the EGF signalling pathway to enhance cell survival during infection.

Previous studies have shown that VGF, which is secreted early during infection, induces continuous activation of the EGFR signalling throughout the virus infection cycle as a consequence of its lower affinity for the receptor (Tzahar *et al*., 1998). This prolonged EGFR activation and signalling would ensure that the infected cell receives a sustained survival signal that antagonizes infection-induced cell death. In accordance with previous studies (Andrade *et al*., 2004), we have shown that triggering signalling pathways by VGF involves a sustained activation of the ERK pathway during infection. Furthermore, our data indicate that activation of the EGFR/MEK pathway is the downstream signalling cascade stimulated by VGF sufficient to elicit a cell survival signal during infection (Figs 5 and 6). Survival pathways stimulated by growth factors in many biological systems are frequently PI3K-Akt-dependent (Cantley, 2002). However, phosphorylation of EGFR is known to only weakly activate the PI3K-Akt pathway (Soltoff *et al*., 1994). Consistent with this we did not observe any changes in activation of the PI3K-Akt pathway during vaccinia infection (Fig. 5A). This is in contrast to the situation observed with several RNA viruses, as well as herpes viruses, which stimulate PI3K-Akt signalling to inhibit apoptosis via intracellular adaptors and decoys (Cooray, 2004).

The role of MEK in EGFR-dependent cell survival has previously been described in keratinocytes, with inhibition of MEK resulting in apoptosis (Jost *et al*., 2001b). In these cells, survival in the absence of matrix-derived signals is dependent on EGFR signalling, which is required to maintain a high-level expression of the anti-apoptotic protein Bcl-xL (Jost *et al*., 2001a). In other epithelial cell types, EGFR-dependent ERK activation is thought to promote cell survival by preventing upregulation of the pro-apoptotic protein Bim (Reginato *et al*., 2003).

Our data reveal that VGF, in concert with F1L, inhibits apoptosis by activating the EGFR/MEK pathway to counteract a cell death pathway principally through the BH3-only protein Bad (Fig. 7). It has been previously reported that during vaccinia infection Bim is a significant inducer of cell death in the absence of F1L (Taylor *et al*., 2006). We found that Bim plays only a modest or redundant role in the induction of cell death by ΔF1L/VGF infection (Fig. 7). Whether these results are attributable to assay/cell type differences or caused by differences between acute siRNA-mediated knockdown versus knockout cells, which may have adapted, is unclear. Therefore, we cannot formally exclude that incomplete Bim knockdown allowed apoptosis to progress as normal, although our findings are consistent with the observation that cell death induced by MVA-ΔF1L infection is not reduced in Bim^–/–^ MEF cells (Fischer *et al*., 2005).

In contrast to Bim, loss of Bad significantly reduced infection-induced cell death by all our virus strains (Fig. 7). It is thought that vaccinia induces apoptosis via several routes (Fischer *et al*., 2005; Taylor and Barry, 2006). For instance, vaccinia dsRNA-induced apoptosis depends exclusively on the BH3-only protein Noxa and is blocked by E3L (Fischer *et al*., 2005). Notably, in the context of ΔE3L infection cell death is not inhibited by the presence of F1L (Fischer *et al*., 2005). Our data indicate that Vaccinia infection, in addition, triggers a Bad-dependent cell death pathway that is antagonized by VGF and F1L. It would be of interest whether VGF and F1L directly inhibit Bad or at some point downstream of Bad to inhibit cell death. Interestingly, no role for Bad in vaccinia-induced cell death has so far been reported. Bad binds to the pro-survival proteins Bcl-2, Bcl-w and Bcl-xL, thus preventing their inhibition of Bak and/or Bax (Chen *et al*., 2005). F1L may inhibit Bad by direct sequestration as observed for Bim (Taylor *et al*., 2006), yet no Bad binding to F1L has been reported thus far. Alternatively, F1L could represent a Bcl-xL-like molecule that retains a high Bak-binding affinity, but unlike host Bcl-xL, does not interact with BH3-only proteins, including Bad. Thus, while activated Bad could sequester Bcl-2 pro-survival proteins to prevent them from neutralizing Bak, F1L would ensure the continued inactivation of Bak. A similar mechanism has been proposed for the myxoma virus protein M11L based on its structure (Kvansakul *et al*., 2007). Interestingly, M11L binding to Bak and Bax is required for its survival promoting activity, yet despite its interaction with Bim, the latter is not involved in the anti-apoptotic function of M11L (Kvansakul *et al*., 2007). While the structure of F1L has been solved (Kvansakul *et al*., 2008), it remains to be established whether a similar mechanism is operating during vaccinia infection. In contrast to M11L, F1L binds Bak but not Bax (Wasilenko *et al*., 2005; Postigo *et al*., 2006). It was recently reported that vaccinia N1L, which structurally resembles Bcl-2 proteins, can interact with Bax and Bad (Cooray *et al*., 2007). It is an intriguing possibility that N1L and F1L target complimentary sets of Bcl-2 proteins, although functionally N1L is not sufficient to inhibit the extensive Bad-dependent cell death induced by the ΔF1L/VGF virus.

Our study has revealed VGF to be an important inhibitor of cell death during infection. It will be interesting to test what targets of the VGF pathway promote survival, including potential phosphorylation of viral proteins required for survival, although our work suggests these would need to be early proteins. In conclusion, we have shown for the first time that a virus is capable of using two cooperative strategies to suppress a Bad-dependent cell death pathway: VGF stimulates EGFR/MEK kinase survival signalling, while F1L inhibits mitochondrial-dependent cell death.

## Experimental procedures

### Viruses and cells

We deleted the F1L locus in the ΔVGF WR strain that lacks both copies of VGF (open reading frames WR009 and WR210) (Buller *et al*., 1988a) using the guanine phosphoribosyl transferase cassette replacement strategy previously used to generate the ΔF1L virus (Postigo *et al*., 2006). The resulting ΔF1L/VGF virus was verified by sequencing and Western blot analysis. Infections of HeLa cells with vaccinia virus and processing for immunoblot analysis were as described previously (Postigo *et al*., 2006).

### Infections and cell survival quantification

HeLa cells plated on dishes coated with fibronectin were infected with WR or the recombinant vaccinia virus strains at a multiplicity of infection of 2 in Opti-MEM reduced serum media and processed for microscopy or immunoblot analysis 20 h post infection. All cell death quantification experiments were performed at least three times, each consisting of a minimum of three counts of over 200 cells each. For western analysis, detached apoptotic cells were collected and added to the adherent cells before cell lysis.

### Drug treatments, transfections and siRNA

HeLa cells were incubated 30 min prior to infection and during infection with the following as appropriate: UO126 (10 μM), PD98059 (36 μM), AG1478 (20 μM), DAPH (15 μM), VGF peptide (10 nM), AraC (50 μM). HeLa cells were transfected with pE/L-MEK1.EE together with pE/L-GFP as a transfection reporter using Effectene according to the manufacturer's instructions (Qiagen) before being infected with WR or the deletion virus strains 2 h later. Cells were processed for immunoblot analysis 20 h after treatments and transfections. HeLa cells were transfected with Bad, Bim or a control non-targeting siRNA (20 nM) according to the manufacturer's instructions using Hiperfect (QIAGEN). One day post transfection, cells were infected with vaccinia virus and incubated for a further 20 h before being processed for Western blotting.

### Antibodies, reagents and chemicals

The following antibodies were used for Western blotting according to standard protocols: ERK1/2, phospho-ERK1/2, Akt, anti-phospho-Akt (Ser473), anti-PARP, anti-active caspase-3 and anti-Bad (Cell Signalling Technology, Beverly, MA); anti-phospho-tyrosine (4G10) was purchased from Upstate and anti-Bim (22–40) was purchased from Calbiochem. Antibodies against the viral proteins A27L, A36R and F1L have been previously described (Rodriguez *et al*., 1985; Röttger *et al*., 1999; Postigo *et al*., 2006). Secondary antibodies for Western blots were goat anti-rabbit or anti-mouse IgG coupled to horseradish peroxidase (Bio-Rad). Validated siRNA for Bad (SI02663003) and Bim (SI02655359) were obtained from Qiagen. siGENOME Non-Targeting siRNA Pool (D-001206-14-20) was obtained from Dharmacon. pEL-MEK.EE was obtained by subcloning from pcDNA3-Rab.MEK.EE (a gift from by Professor P.C. Clarke, Dundee University, Dundee, UK). UO126 was purchased from Promega, AG1478 was obtained from Merck. PD98059 was from Calbiochem and cytosine arabinoside (AraC) was obtained from Sigma. Residues 38–88 of vaccinia WR VGF, corresponding to the EGF-like domain as defined by Tzahar *et al*. (1998), were synthesized by Cancer Research UK.
